# Supplementary material for: Immunoglobulin G N-glycan markers of accelerated biological aging during chronic HIV infection
Source: Nat Commun. 2024 Apr 10;15:3035. doi: 10.1038/s41467-024-47279-4 (PMC11006954; doi:10.1038/s41467-024-47279-4)
Supplement: Supplementary file 1 — Supplementary Information [file 41467_2024_47279_MOESM1_ESM.pdf]

Supplementary Information

Immunoglobulin G *N*-glycan Markers of Accelerated Biological Aging During Chronic HIV Infection

Leila B Giron[1], Qin Liu[1], Opeyemi S Adeniji[1], Xiangfan Yin[1], Toshitha Kannan[1], Jianyi Ding[1], David Y. Lu[1,2], Susan Langan[3], Jinbing Zhang[3], Joao L. L. C. Azevedo[1], Shuk Hang Li[4], Sergei Shalygin[5], Parastoo Azadi[5], David B Hanna[6], Igho Ofotokun[7], Jason Lazar[8], Margaret A. Fischl[9], Sabina Haberlen[3], Bernard Macatangay[10], Adaora A. Adimora[11], Beth D. Jamieson[12], Charles Rinaldo[10], Daniel Merenstein[13], Nadia R. Roan[14,15], Olaf Kutsch[16], Stephen Gange[3], Steven M. Wolinsky[17], Mallory D. Witt[18], Wendy S. Post[3], Andrew Kossenkov[1], Alan L. Landay[19], Ian Frank[4], Phyllis C. Tien[15], Robert Gross[4], Todd T. Brown[3], Mohamed Abdel-Mohsen[1†]

[1]The Wistar Institute, Philadelphia, PA, USA; [2]Cornell University, New York, NY, USA; [3]Johns Hopkins University, Baltimore, MD, USA; [4]University of Pennsylvania Perelman School of Medicine, Philadelphia, PA, USA; [5]University of Georgia, Athens, Georgia, USA; [6]Albert Einstein College of Medicine, Bronx, NY, USA; [7]Division of Infectious Diseases, Department of Medicine, Emory University School of Medicine, Atlanta, GA, USA; [8]SUNY Downstate Health Sciences University, New York, NY, USA; [7]Division of Infectious Disease, Department of Medicine, University of Miami, Miami, FL, USA; [10]University of Pittsburgh, Pittsburgh, PA, USA; [11]University of North Carolina, Chapel Hill, NC, USA; [12]University of California, Los Angeles, Los Angeles, CA, USA; [13]Georgetown University Medical Center, Washington, DC, USA; [14]Gladstone Institutes, San Francisco, CA, USA; [15]University of California San Francisco, San Francisco, CA, USA; [16]University of Alabama at Birmingham, Birmingham, Alabama, USA; [17]Northwestern University, Chicago, IL, USA; [18]Lundquist Institute of Biomedical Research at Harbor-UCLA Medical Center, Torrance, CA, USA; [19]Rush University, Chicago, IL, USA.

[†]Corresponding author: Mohamed Abdel-Mohsen, Ph.D. Associate Professor, Vaccine and Immunotherapy Center, The Wistar Institute. 3601 Spruce Street, Philadelphia, PA 19104. Phone: 215-898-6008. Email: mmohsen@Wistar.org

a

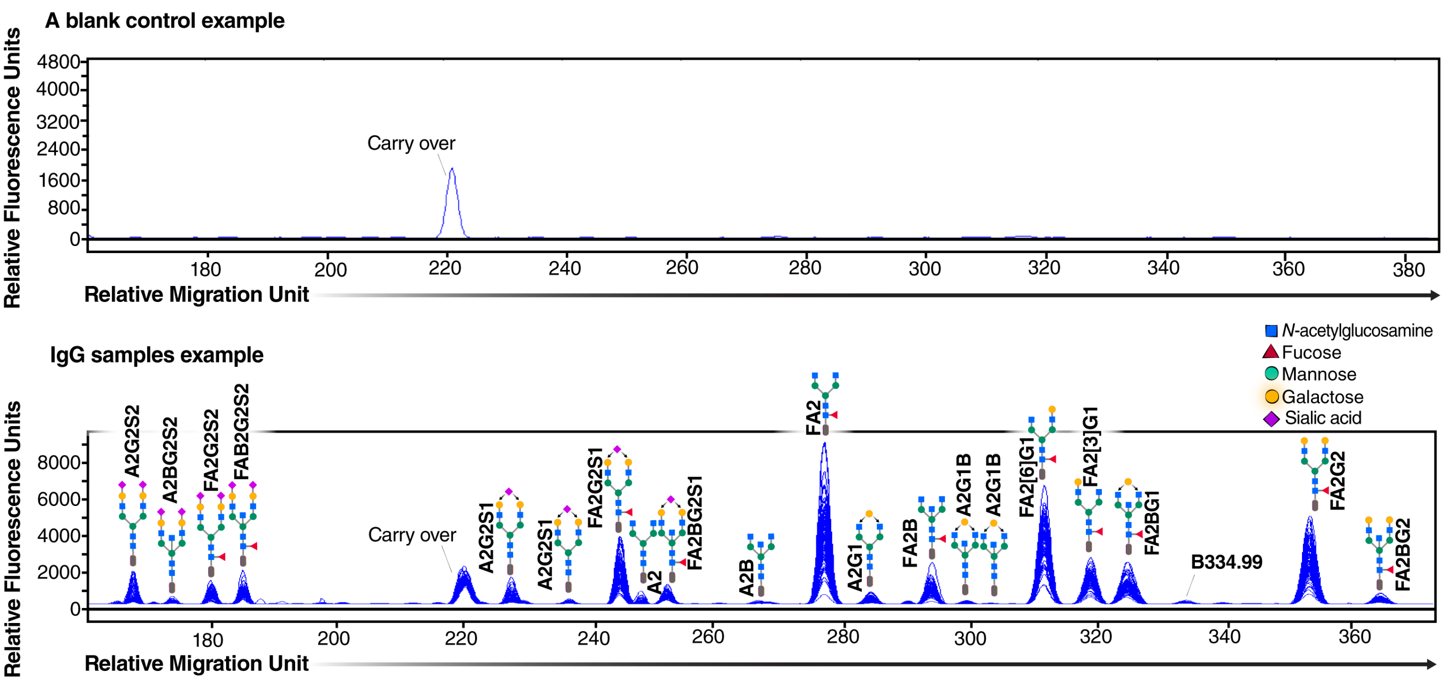

b

IgG Glycosylation group	Glycan traits
**Total sialylated group**	A2G2S1 + A2G2S1 + FA2G2S1 + FA2BG2S1 + A2G2S2 + A2BG2S2 + FA2G2S2 + FA2BG2S2
**Agalactosylated group**	A2 + A2B + FA2 + FA2B
**Total terminal galactosylated**	A2G2S1 + A2G2S1 + FA2G2S1 + FA2BG2S1 + A2G1 + A2G1B + A2G1B + FA2[3]G1 + FA2[6]G1 + FA2BG1 + FA2G2 + FA2BG2
**Fucosylated group**	FA2G2S2 + FA2BG2S2 + FA2G2S1 + FA2BG2S1 + FA2 + FA2B + FA2[3]G1 + FA2[6]G1 + FA2BG1 + FA2G2 + FA2BG2
**Bisected Group**	A2BG2S2 + FA2BG2S2 + FA2BG2S1 + A2B + FA2B + A2G1B + A2G1B + FA2BG1 + FA2BG2

c

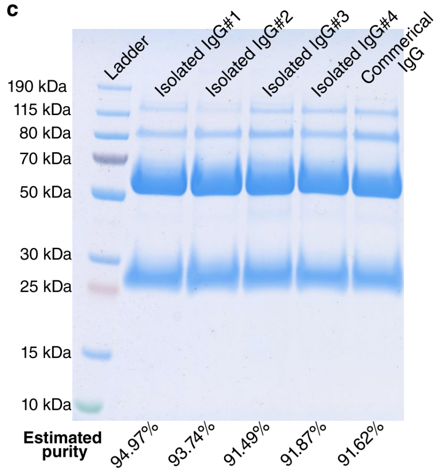

d

Liquid chromatography-tandem mass spectrometry (LC-MS/MS) analysis

Name of protein	Commercial IgG	Participant IgG
Immunoglobulin G	99.14%	97.93%
Albumin	0.66%	0.63%
C4b-binding protein alpha chain	0.00%	0.40%
Fibronectin;Anastellin;Ugl-Y1;Ugl-Y2;Ugl-Y3	0.00%	0.30%
Ig-like domain-containing protein (Fragment)	0.09%	0.12%
Complement C4-A	0.03%	0.16%
Serotransferrin	0.01%	0.001%

e

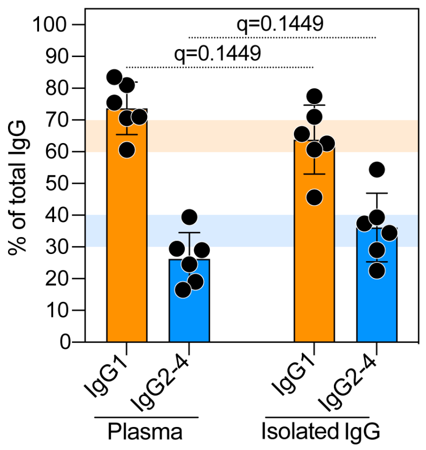

Supplementary Figure 1. (a) Schematic of IgG glycan analysis by capillary electrophoresis. **(b)** Illustration of the various glycan traits included in the IgG *N*-glycan groups. **(c)** SDS-PAGE illustrating the purity of four randomly selected IgG isolations along with a commercially available IgG. **(d)** Liquid chromatography-tandem mass spectrometry (LC-MS/MS) analysis of a randomly selected IgG samples and commercially available IgG. **(e)** Comparisons of the percentages of IgG1 and the combination of IgG2, IgG3, and IgG4 in six randomly selected plasma samples and their parallel isolated IgG, determined by ELISA. Statistical analysis of these comparisons was conducted using one-way ANOVA, adjusted using the Benjamini & Hochberg method. Source data are provided as a Source Data file.

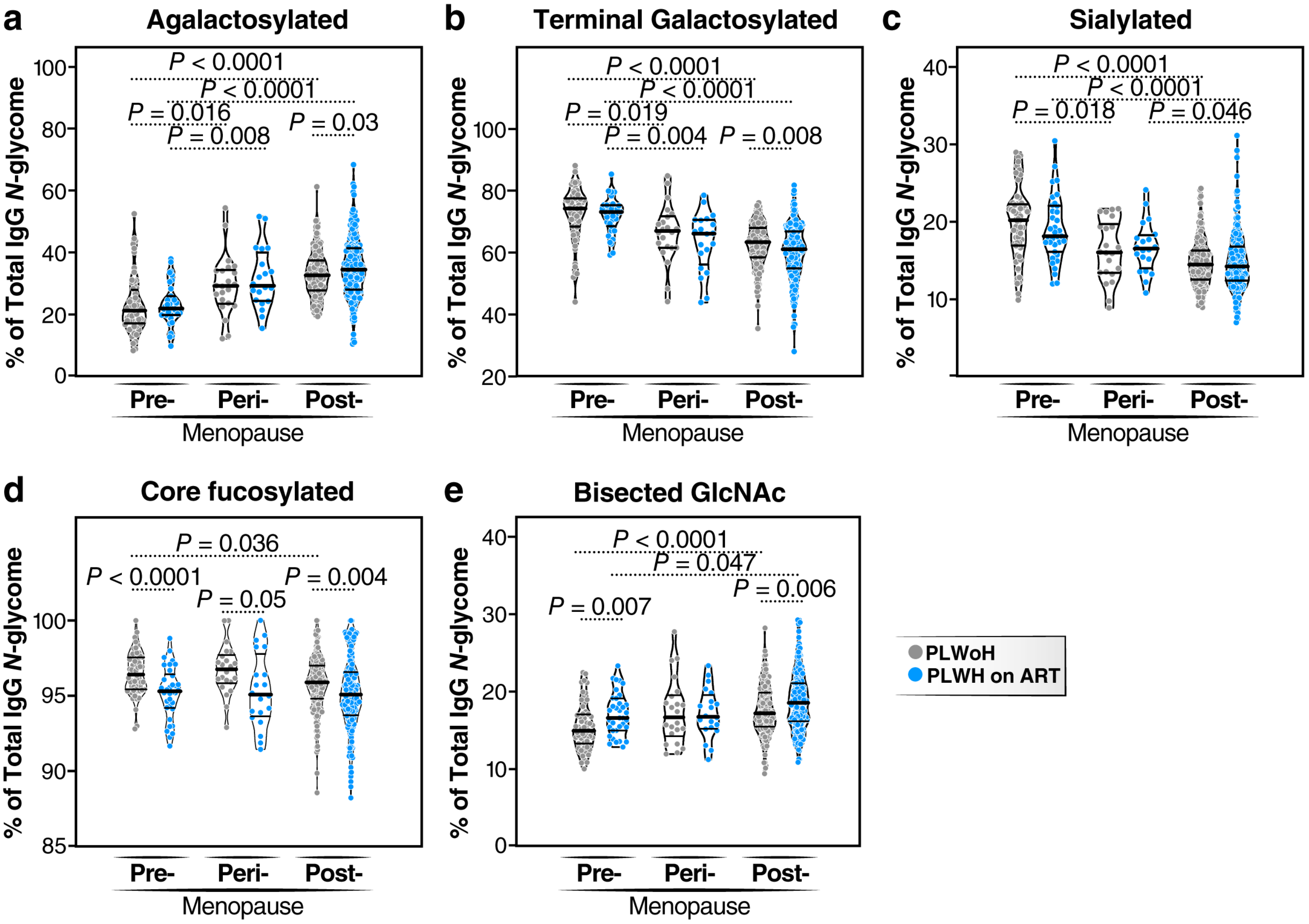

Supplementary Figure 2. Impact of menopause/age on IgG glycans during ART-suppressed HIV infection. Two-tailed Kruskal-Wallis comparisons of IgG *N*-glycan groups between pre-, peri-, and post-menopause women living with or without HIV. Violin plots depict median and IQR. N=487 biological samples. Source data are provided as a Source Data file.

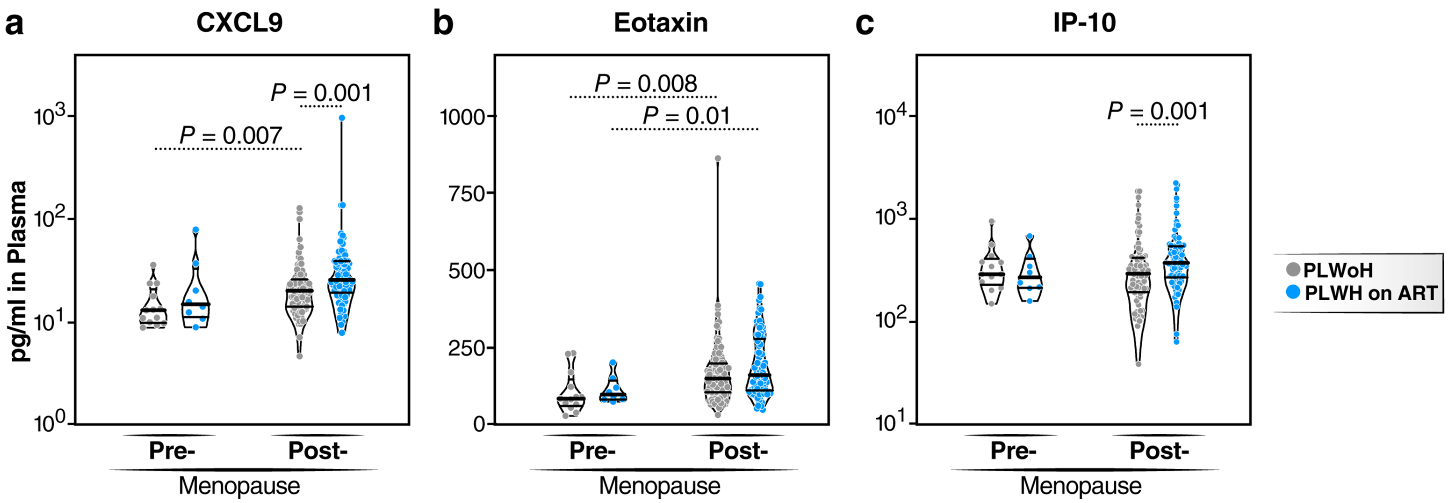

Supplementary Figure 3. Impact of menopause/age on inflammation during ART-suppressed HIV infection. Two-tailed Kruskal-Wallis comparisons of inflammation markers between pre-, peri-, and post-menopause women living with or without HIV. Violin plots illustrate the median and IQR. N=189 biological samples. Source data are provided as a Source Data file.

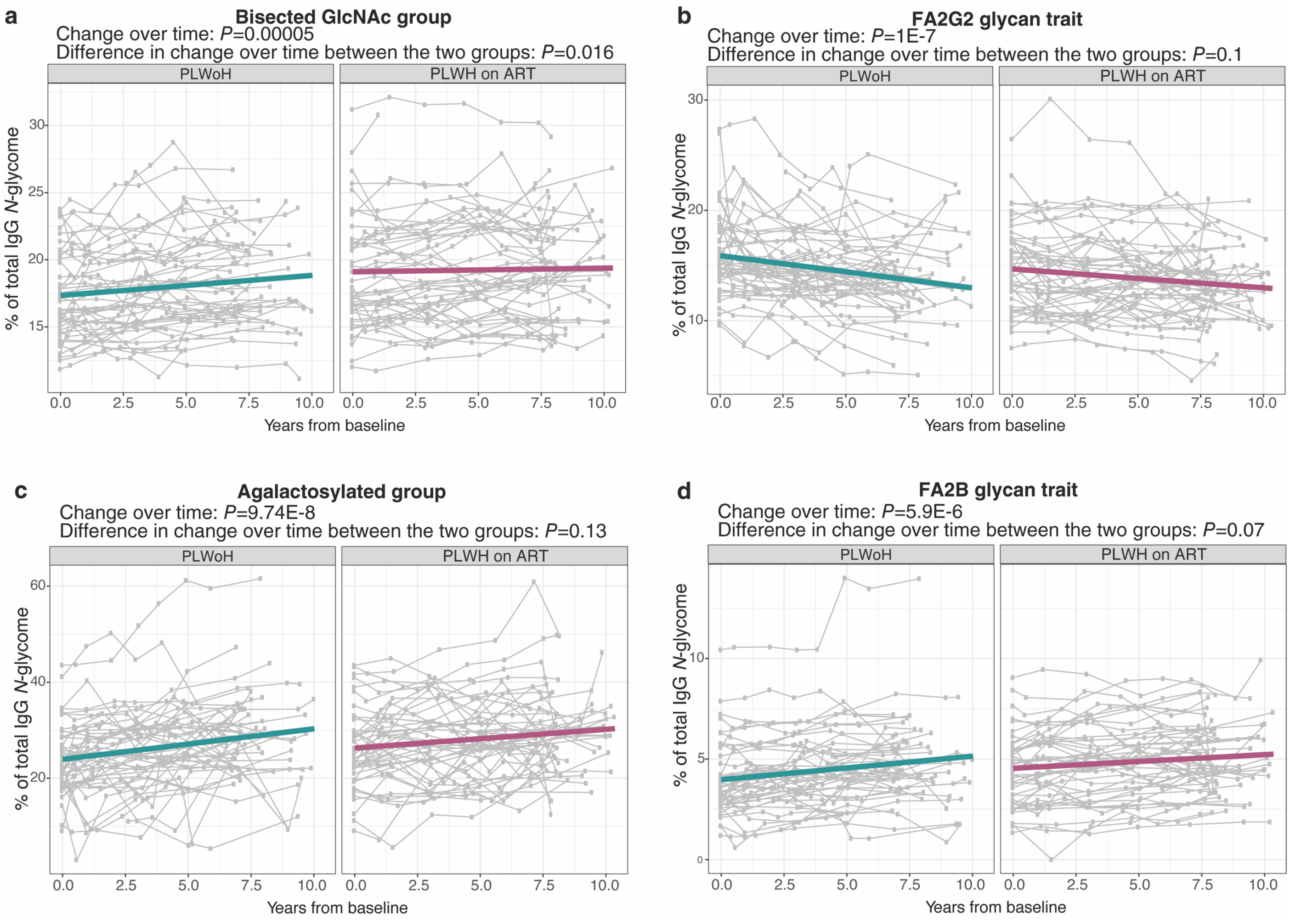

Supplementary Figure 4. Longitudinal analysis of IgG glycans in PLWH and PLWoH. (a-d) Examples of the analysis of IgG glycans over time in PLWH and their age-, sex-, ethnicity-, and BMI-matched PLWoH. The significance of change over time and the difference in change over time between the groups were determined using multivariable mixed effects models. N=622 biological samples from 94 people living with or without HIV. Source data are provided as a Source Data file.

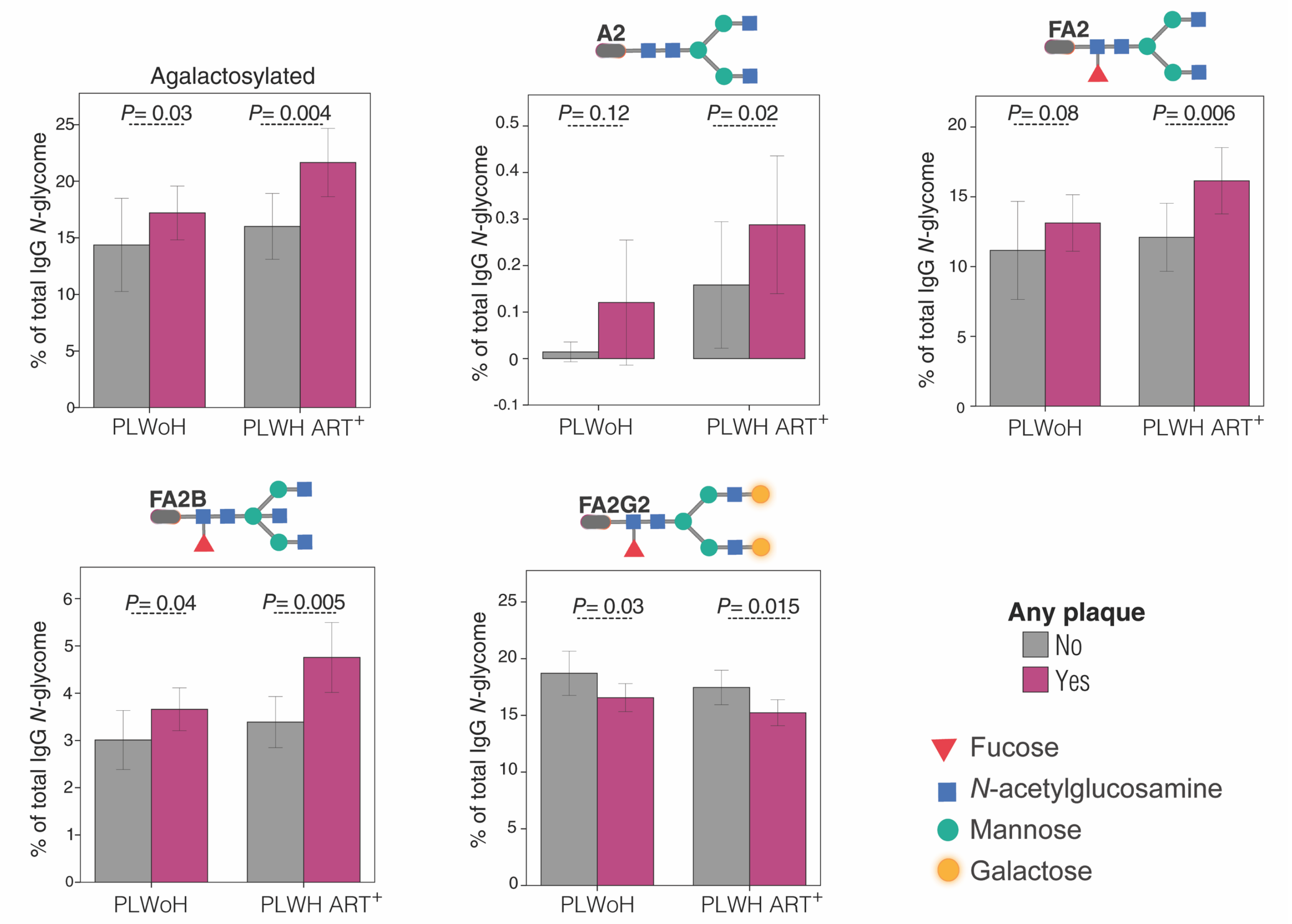

Supplementary Figure 5. Association of IgG glycans with the incidence of coronary plaques in PLWH on ART. Two-tailed Mann-Whitney tests were performed between individuals living with or without HIV, stratified by the presence or absence of any coronary plaques, to examine the relationship between IgG glycans and plaque incidence. Error bars represent Mean with 95% CI. N=112 biological samples. Source data are provided as a Source Data file.

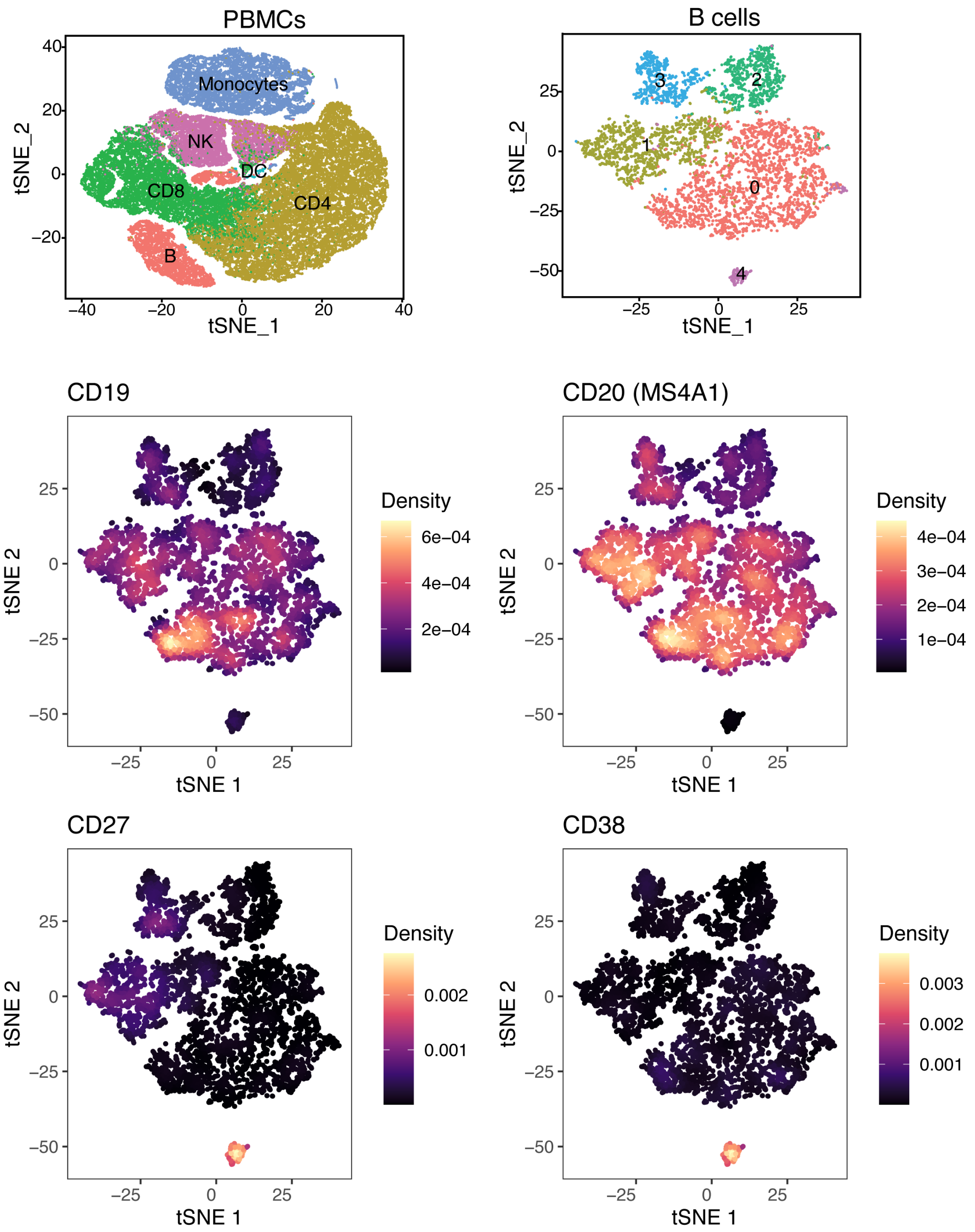

Supplementary Figure 6: Expression of key B cell markers in B cell clusters. t-SNE plots displaying: PBMCs and B cell clusters from the single-cell cite-seq experiments as well as the gene expression densities of CD19, CD20, CD27, and CD38 in the various B cell clusters observed from the single-cell CITE-seq experiments. The two cluster on the top did not express high levels of the B cell markers, CD19 and CD20 and were excluded from the analysis.

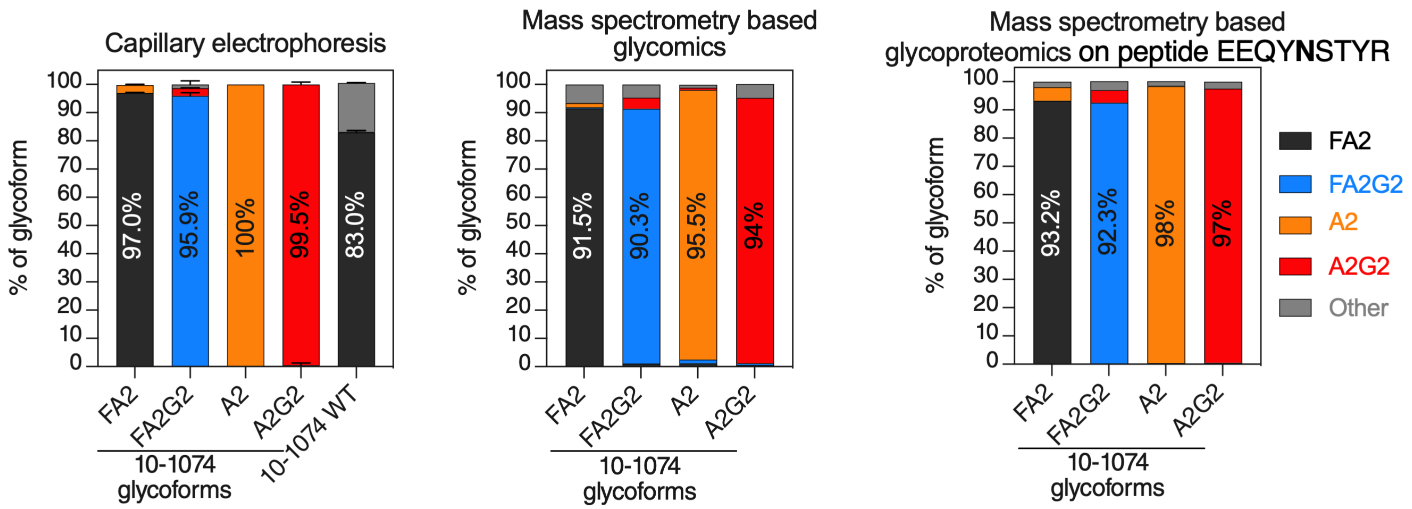

Supplementary Figure 7: Purity of the 10-1074 glycoforms. Percentage of glycans within each glycoform and wild-type 10-1074 assessed using capillary electrophoresis in triplicate (left), mass spectrometry based glycomics (middle), and mass spectrometry based glycoproteomics (right). Source data are provided as a Source Data file.

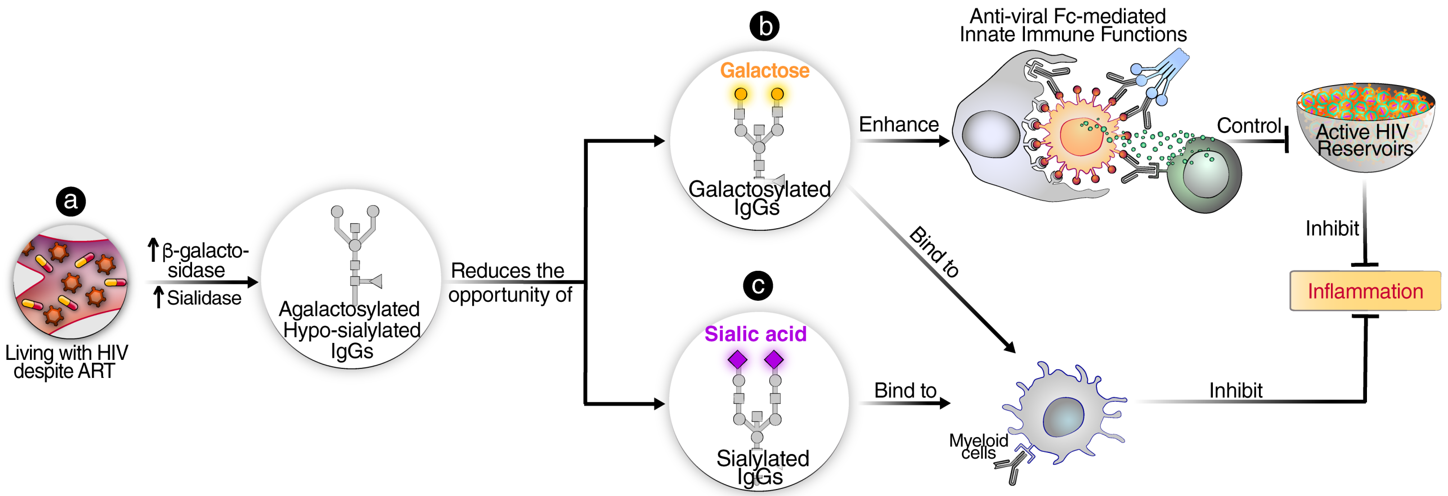

Supplementary Figure 8. A proposed model on how HIV-associated loss of IgG galactose and sialic Acid can lead to inflammation in PLWH. (a) Living with HIV is associated with a reduction in IgG galactosylation and sialylation, leading to an accumulation of agalactosylated and hyposialylated IgGs. This alteration in IgG galactosylation and sialylation may lead to inflammation by diminishing several anti-viral and anti-inflammatory functions of galactose and sialic acid on IgGs. Specifically: **(b)** Galactosylated glycans enhance anti-HIV Fc-mediated immune function, potentially resulting in smaller active HIV reservoirs and reduced inflammation. Moreover, IgG galactose facilitates interactions between FcγRIIB (CD32b) and dectin-1, triggering anti-inflammatory cascades in myeloid cells. **(c)** Sialylated glycans play a role in reducing inflammation by binding to specific anti-inflammatory receptors on myeloid cells. The HIV-associated reduction in galactose and sialic acid can lead to inflammation by impairing the potential for eliciting these vital antiviral and anti-inflammatory functions of IgGs.

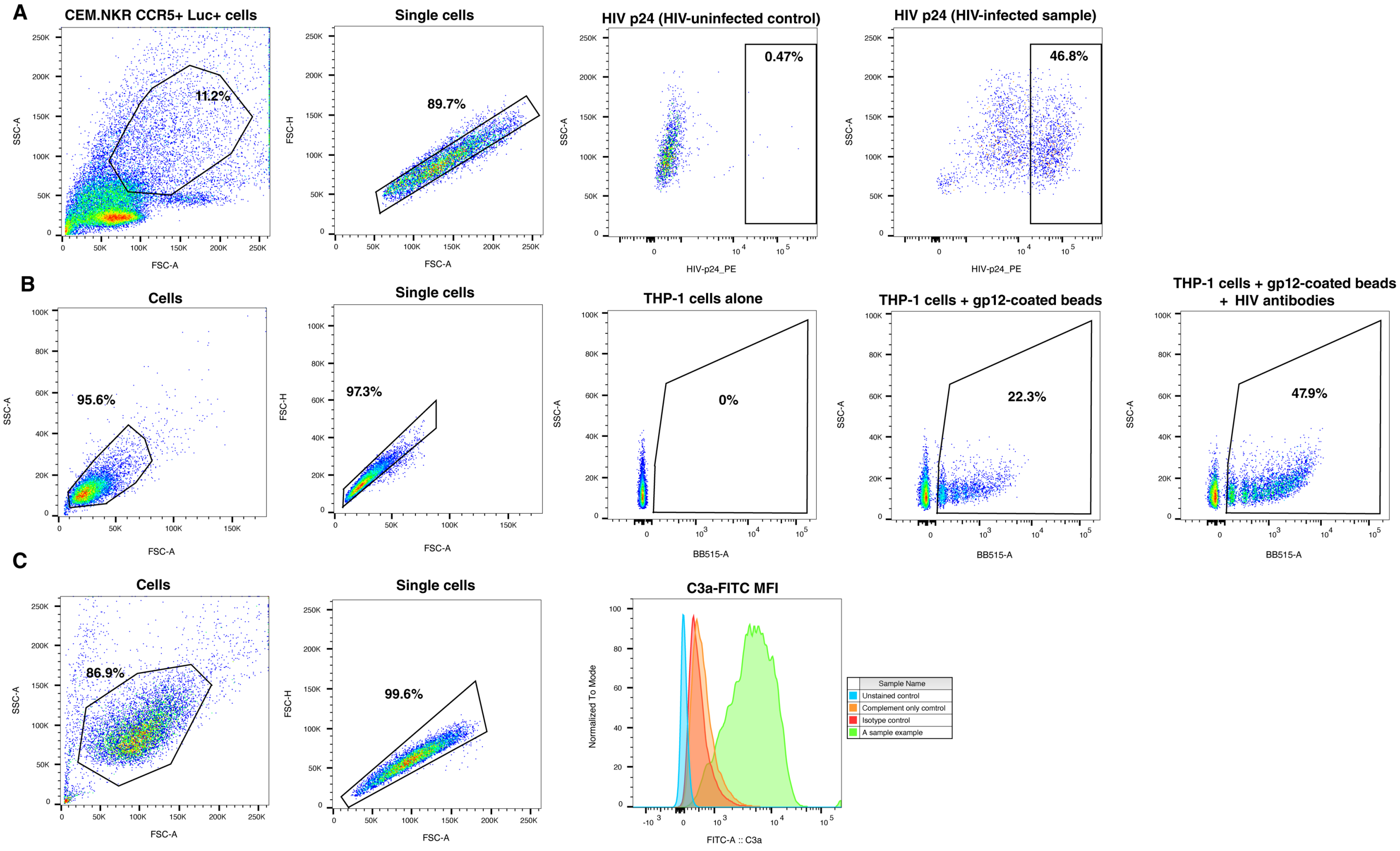

Supplementary Figure 9. Gating strategies for: (a) the p24 intracellular staining as part of the antibody-dependent cell-mediated cytotoxicity (ADCC) assays using glyco-engineered antibodies; **(b)** antibody-dependent cellular phagocytosis (ADCP) assays; and **(c)** Antibody-dependent complement deposition (ADCD) assays.

Supplementary Table 1. IgG *N*-glycan alterations associated with living with HIV and/or ART use.

Variable	Group	Women				Men				Interaction between HIV and gender		Notes
		N	Mean (SD) %	P	FDR	N	Mean%	P	FDR	Estimate of mean (SD) difference	p value	
A2 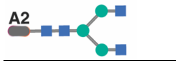	PLWoH	235	0.23 (0.33)	0.0000004	0.000004	253	0.27 (0.34)	1.65186E-10	6.9378E-10	0.17 (0.06)	0.01	Glycan traits that their levels increase in PLWH on ART, than controls, but their levels increase more in men than women
	PLWH on ART	254	0.46 (0.59)			243	0.58 (0.61)					
A2BG1 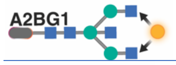	PLWoH	235	0.13 (0.18)	0.002	0.005	253	0.20 (0.20)	1.56538E-13	1.09577E-12	0.15 (0.03)	0.000005	
	PLWH on ART	254	0.2 (0.23)			243	0.38 (0.30)					
A2G1 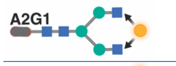	PLWoH	235	0.70 (0.57)	0.000001	0.00001	253	0.95 (0.67)	2.58771E-11	1.35855E-10	0.25 (0.10)	0.01	
	PLWH on ART	254	1.01 (0.74)			243	1.43 (0.83)					
A2G2S1 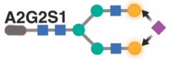	PLWoH	235	0.25 (0.18)	0.00002	0.00012	253	0.29 (0.23)	3.14439E-15	3.30161E-14	0.07 (0.03)	0.02	
	PLWH on ART	254	0.32 (0.23)			243	0.42 (0.20)					
FA2BG1 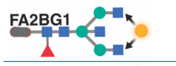	PLWoH	235	5.89 (1.47)	0.002	0.005	253	6.20 (1.59)	0.0000001	0.0000002	0.62 (0.21)	0.002887	
	PLWH on ART	254	6.34 (1.57)			243	6.99 (1.68)					
A2BG2S2 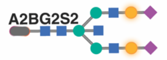	PLWoH	235	0.19 (0.14)	0.0001	0.0004	253	0.33 (0.36)	0.0000005	0.0000009	0.01 (0.04)	0.68	Glycan traits that their levels increase in PLWH on ART, than controls.
	PLWH on ART	254	0.24 (0.19)			243	0.40 (0.24)					
FA2B 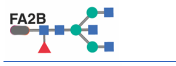	PLWoH	235	4.80 (2.04)	0.002	0.005	253	4.18 (1.83)	0.0000003	0.0000007	0.46 (0.25)	0.06	
	PLWH on ART	254	5.45 (2.35)			243	4.93 (1.79)					
FA2 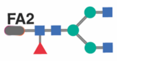	PLWoH	235	24.57 (7.47)	0.005	0.008	253	20.37 (6.29)	0.0001	0.0001	0.67 (0.93)	0.47	
	PLWH on ART	254	26.79 (8.33)			243	22.65 (6.54)					
FAB2G2S2 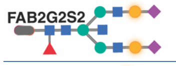	PLWoH	235	2.28 (0.55)	0.041	0.057	253	2.78 (1.59)	0.30	0.35	-0.59 (0.14)	0.000025	Glycan traits that their levels increase in women living with HIV, than controls
	PLWH on ART	254	2.42 (0.67)			243	2.32 (0.78)					
A2G2S2 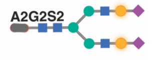	PLWoH	235	0.94 (0.41)	0.0004	0.001	253	1.80 (1.30)	0.43	0.45	-0.12 (0.14)	0.40	
	PLWH on ART	254	1.03 (0.37)			243	1.9 (1.52)					
A2B 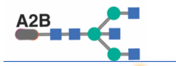	PLWoH	235	0.3 (0.26)	0.108	0.142	253	0.29 (0.26)	7.88248E-09	2.36475E-08	0.15 (0.04)	0.000139	Glycan traits that their levels increase in men living with HIV, than controls
	PLWH on ART	254	0.35 (0.33)			243	0.45 (0.33)					
A2G2S1 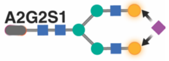	PLWoH	235	0.69 (0.46)	0.506	0.591	253	1.23 (0.85)	0.000046	0.000081	0.22 (0.10)	0.03	
	PLWH on ART	254	0.72 (0.50)			243	1.57 (1.01)					
FA2BG2S1 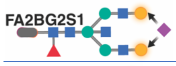	PLWoH	235	7.99 (3.13)	0.0001	0.0004	253	8.84 (3.33)	1.22464E-09	4.28624E-09	-1.00 (0.36)	0.01	Glycan traits that their levels decrease in PLWH on ART, than controls, but their levels decrease more in men than women
	PLWH on ART	254	6.90 (2.60)			243	7.11 (2.37)					
FA2[3]G1 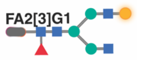	PLWoH	235	9.02 (1.41)	4.69969E-17	9.86935E-16	253	9.64 (1.64)	3.16271E-18	6.64169E-17	-0.07 (0.20)	0.73	Glycan traits that their levels decrease in PLWH on ART, than controls.
	PLWH on ART	254	7.94 (1.34)			243	8.37 (1.48)					
FA2G2 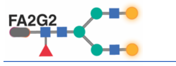	PLWoH	235	14.41 (4.50)	0.002	0.005	253	15.36 (3.56)	0.0000002	0.0000005	-0.79 (0.49)	0.11	
	PLWH on ART	254	13.04 (4.20)			243	13.70 (3.45)					
FA2[6]G1 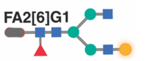	PLWoH	235	21.94 (2.17)	0.006	0.010	253	20.25 (2.61)	0.48	0.48	0.55 (0.32)	0.08	Glycan traits that their levels decrease in women living with HIV on ART, than controls.
	PLWH on ART	254	21.19 (2.41)			243	20.48 (2.68)					
FA2G2S2 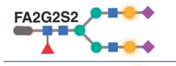	PLWoH	235	1.74 (0.66)	0.433	0.535	253	2.28 (1.25)	0.003	0.005	-0.61 (0.11)	0.0000001	Glycan traits that their levels decrease in men living with HIV on ART, than controls.
	PLWH on ART	254	1.79 (0.63)			243	1.83 (0.64)					
FA2BG2 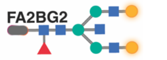	PLWoH	235	1.34 (0.48)	0.662	0.696	253	1.58 (0.50)	0.23	0.29	0.03 (0.06)	0.59	Glycan traits that their levels do not change in PLWH on ART, than controls.
	PLWH on ART	254	1.30 (0.52)			243	1.55 (0.44)					
FA2BG2S1 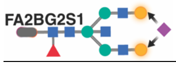	PLWoH	235	2.03 (0.51)	0.655	0.696	253	2.56 (1.07)	0.05	0.07	-0.31 (0.10)	0.002791	
	PLWH on ART	254	2.03 (0.61)			243	2.28 (0.68)					
A2BG1 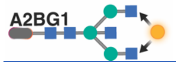	PLWoH	235	0.05 (0.08)	0.801	0.801	253	0.05 (0.08)	0.12	0.16	-0.01 (0.01)	0.33	
	PLWH on ART	254	0.05 (0.07)			243	0.04 (0.07)					

■ *N*-acetylglucosamine ▲ Fucose ● Mannose ● Galactose ◆ Sialic acid

Two-tailed unpaired t-tests and false discovery rates (FDRs) were calculated to account for multiple tests over studied markers. Interaction P values were calculated using multivariable models, adjusting for age, ethnicity, and BMI.

Supplementary Table 2. Comparisons of IgG glycans among pre-menopause, peri-menopause, and post-menopause women.

	**Pre-menopause**			**Early/Late peri-menopause**			**Post-menopause**			**PLWoH pre-menoapuse**		**PLWH on ART pre-menoapuse**	
Glycan Trait	PLWoH	PLWH on ART		PLWoH	PLWH on ART		PLWoH	PLWH on ART		*vs* PLWoH peri-menopause	*vs* PLWoH Post-menopause	*vs* PLWH on ART peri-menopause	*vs* PLWH on ART Post-menopause
	Median	Median	*P* value	Median	Median	*P* value	Median	Median	*P* value	*P* value	*P* value	*P* value	*P* value
A2G2S2	0.94%	0.93%	0.55	0.86%	1.20%	**0.018**	0.88%	0.97%	**0.002**	KW non-significant		KW non-significant	
A2BG2S2	0.18%	0.29%	**0.002**	0.08%	0.29%	**0.016**	0.22%	0.25%	0.054	0.834	0.200	KW non-significant	
FA2G2S2	2.15%	2.04%	0.305	1.67%	1.97%	0.137	1.52%	1.64%	**0.04**	**0.009**	**<0.0001**	1.000	**0.011**
FA2BG2S2	2.17%	2.38%	0.321	2.15%	2.44%	0.200	2.22%	2.26%	0.12	KW non-significant		KW non-significant	
A2G2S1	0.87%	0.97%	0.790	0.62%	0.93%	0.124	0.59%	0.69%	0.287	0.067	**<0.0001**	1.000	**0.007**
A2G2S1	0.21%	0.40%	**0.001**	0.19%	0.37%	**0.007**	0.31%	0.34%	**0.033**	1.000	**0.015**	KW non-significant	
FA2G2S1	11.09%	9.51%	**0.042**	8.55%	7.18%	0.365	6.52%	5.90%	**0.026**	**0.045**	**<0.0001**	**0.018**	**<0.0001**
A2	0.00%	0.11%	**0.002**	0.00%	0.00%	0.223	0.19%	0.40%	**0.001**	0.900	**<0.0001**	1.000	**0.002**
FA2BG2S1	2.04%	1.99%	0.436	1.91%	1.92%	0.821	1.95%	1.95%	0.89	KW non-significant		KW non-significant	
FA2	0.00%	0.18%	0.578	0.00%	0.31%	0.317	0.39%	0.43%	0.6	1.000	**<0.0001**	0.800	**0.007**
G0F	17.10%	18.54%	0.547	22.98%	24.08%	0.529	26.29%	27.09%	0.09	**0.025**	**<0.0001**	**0.009**	**<0.0001**
A2G1	0.54%	1.09%	**<0.0001**	0.60%	0.75%	0.224	0.66%	0.86%	**0.006**	KW non-significant		KW non-significant	
FA2B	2.97%	3.25%	0.305	4.51%	4.63%	0.880	4.80%	5.46%	0.069	**0.004**	**<0.0001**	**0.037**	**<0.0001**
A2BG1	0.00%	0.18%	**0.040**	0.00%	0.00%	0.110	0.11%	0.17%	0.198	0.900	**0.040**	KW non-significant	
A2BG1	0.00%	0.00%	0.548	0.00%	0.00%	0.600	0.00%	0.00%	0.647	KW non-significant		KW non-significant	
FA2[6]G1	21.67%	21.81%	0.975	21.37%	21.84%	0.650	21.95%	21.36%	**0.004**	KW non-significant		KW non-significant	
FA2[3]G1	8.73%	8.10%	0.137	8.92%	8.14%	0.160	9.27%	7.87%	**<0.0001**	KW non-significant		KW non-significant	
FA2BG1	5.85%	6.48%	**0.006**	6.06%	5.86%	0.920	5.79%	6.17%	**0.018**	KW non-significant		KW non-significant	
FA2G2	18.96%	17.74%	0.300	14.41%	14.27%	0.670	12.60%	11.85%	0.074	**0.014**	**<0.0001**	**0.006**	**<0.0001**
FA2BG2	1.58%	1.62%	0.123	1.40%	1.39%	0.400	1.25%	1.22%	0.947	1.000	**<0.0001**	**0.007**	**<0.0001**

KW = Kruskal Wallis tests

Supplementary Table 3. Correlations between IgG glycans and chronological age.

	Variable	Group	Women Spearman's *rho*	Women *P*	Women Slope	Women Slope *P*	Men Spearman's *rho*	Men *P*	Men Slope	Men Slope *P*	All Spearman's *rho*	All *P*	All Slope	All Slope *P*
Individual glycans	A2	PLWoH	0.350	**<0.0001**	0.0130	***0.050***	0.394	**<0.0001**	0.0149	0.120	0.380	**<0.0001**	0.0140	**0.008**
		PLWH on ART	0.310	**<0.0001**	0.0250		0.347	**<0.0001**	0.0230		0.338	**<0.0001**	0.0247	
	A2BG1	PLWoH	0.079	0.2300	0.0014	0.887	0.089	0.1600	0.0009	0.660	0.778	0.0800	0.0010	0.786
		PLWH on ART	0.110	0.0800	0.0013		0.184	**0.0040**	0.0010		0.145	**0.0012**	0.0012	
	A2G1	PLWoH	0.142	**0.0300**	0.0114	0.265	0.320	**<0.0001**	0.2490	0.150	0.272	**<0.0001**	0.2150	0.077
		PLWH on ART	-0.001	0.9900	0.0020		0.140	**0.0350**	0.0120		0.090	**0.0460**	0.0100	
	A2G2S1	PLWoH	-0.240	**0.0002**	-0.0170	0.200	-0.070	0.2600	-0.0100	***0.026***	-0.030	0.4000	-0.0038	***0.045***
		PLWH on ART	-0.130	**0.0350**	-0.0095		-0.300	**<0.0001**	-0.0345		0.160	**0.0003**	-0.0180	
	FA2BG1	PLWoH	0.023	0.7260	0.0040	0.600	0.360	**<0.0001**	0.0700	***0.030***	0.220	**<0.0001**	0.0400	0.078
		PLWH on ART	-0.009	0.8800	-0.0060		0.120	0.0580	0.0300		0.068	0.1290	0.0200	
	A2BG2S2	PLWoH	0.220	**0.0008**	0.0046	***0.018***	0.040	0.5550	0.0030	***0.080***	0.200	**<0.0001**	0.0060	***0.002***
		PLWH on ART	-0.005	0.9400	-0.0004		-0.090	0.1600	-0.0030		-0.021	0.6400	-0.0010	
	FA2B	PLWoH	0.500	**<0.0001**	0.1200	0.710	0.350	**<0.0001**	0.0900	0.400	0.370	**<0.0001**	0.0900	0.690
		PLWH on ART	0.430	**<0.0001**	0.1300		0.270	**<0.0001**	0.0700		0.340	**<0.0001**	0.0900	
	FA2	PLWoH	0.460	**<0.0001**	0.4560	0.769	0.210	**0.0008**	0.1900	0.640	0.250	**<0.0001**	0.2200	0.180
		PLWH on ART	0.380	**<0.0001**	0.4800		0.240	**0.0002**	0.2200		0.290	**<0.0001**	0.3000	
	FAB2G2S2	PLWoH	-0.020	0.9700	-3.9E-06	0.820	-0.03	0.6200	0.0060	0.970	-0.005	0.9000	0.0050	0.230
		PLWH on ART	-0.050	0.4260	-0.0010		-0.062	0.3300	-0.0060		-0.069	0.1250	-0.0049	
	A2G2S2	PLWoH	-0.089	0.1750	-0.0010	0.490	-0.270	**<0.0001**	-0.0400	0.580	-0.555	0.2200	-0.0050	0.440
		PLWH on ART	-0.070	**0.0246**	-0.0050		-0.170	**0.0060**	-0.0300		-0.100	**0.0400**	-0.0100	
	A2B	PLWoH	0.411	**<0.0001**	0.0140	0.830	0.330	**<0.0001**	0.0100	0.380	0.350	**<0.0001**	0.0110	0.260
		PLWH on ART	0.280	**<0.0001**	0.0137		0.320	**<0.0001**	0.0130		0.310	**<0.0001**	0.0140	
	A2G2S1	PLWoH	0.260	**<0.0001**	0.0070	***0.003***	0.150	**0.0200**	0.0040	***0.039***	0.210	**<0.0001**	0.2000	***0.001***
		PLWH on ART	-0.010	0.8450	-0.0010		-0.010	0.8800	-0.0010		0.001	0.9750	-0.0013	
	FA2G2S1	PLWoH	-0.508	**<0.0001**	-0.2370	0.119	-0.265	**<0.0001**	-0.1060	0.420	-0.330	**<0.0001**	-0.1400	0.440
		PLWH on ART	-0.449	**<0.0001**	-0.1800		-0.260	**<0.0001**	-0.0800		-0.350	**<0.0001**	-0.1200	
	FA2[3]G1	PLWoH	-0.030	0.6710	-0.0110	0.096	-0.180	**0.0060**	-0.0600	0.990	-0.180	**<0.0001**	-0.0640	0.947
		PLWH on ART	-0.170	**0.0060**	-0.0600		-0.160	**0.0100**	-0.0600		-0.170	**0.0002**	-0.0660	
	FA2G2	PLWoH	-0.511	**<0.0001**	-0.3300	0.770	-0.250	**<0.0001**	-0.1300	0.630	-0.340	**<0.0001**	-0.1900	0.460
		PLWH on ART	-0.490	**<0.0001**	-0.3200		-0.340	**<0.0001**	-0.1500		-0.410	**<0.0001**	-0.2170	
	FA2[6]G1	PLWoH	-0.026	0.6900	-0.0020	0.260	-0.100	0.1280	-0.0187	***0.007***	-0.020	0.6400	0.0002	0.400
		PLWH on ART	0.140	**0.0300**	-0.0200		-0.180	**0.0060**	0.0300		0.040	0.3800	0.0100	
	FA2G2S2	PLWoH	-0.380	**<0.0001**	-0.0360	0.388	-0.260	**<0.0001**	-0.0300	0.310	-0.250	**<0.0001**	-0.0230	0.870
		PLWH on ART	-0.300	**<0.0001**	-0.0300		-0.250	**<0.0001**	-0.0200		-0.270	**<0.0001**	-0.0240	
	FA2BG2	PLWoH	-0.250	**0.0001**	-0.0100	0.170	0.080	0.1900	0.0030	0.140	-0.008	0.8600	-0.0005	***0.016***
		PLWH on ART	-0.310	**<0.0001**	-0.0200		-0.110	0.0800	-0.0046		-0.190	**<0.0001**	-0.0100	
	FA2BG2S1	PLWoH	-0.070	0.3100	-0.0040	0.957	-0.060	0.3000	-0.0070	0.440	0.005	0.9000	0.0040	0.070
		PLWH on ART	-0.008	0.2100	-0.0030		-0.210	**0.0085**	-0.0150		-0.130	**0.0040**	-0.0080	
	A2BG1	PLWoH	0.220	**0.0005**	0.0050	0.140	0.350	**<0.0001**	0.0080	0.630	0.320	**<0.0001**	0.0800	0.330
		PLWH on ART	0.050	0.4080	0.0010		0.200	**0.0020**	0.0070		0.150	**0.0010**	0.0060	
Grouped glycans	**Bisected**	PLWoH	0.300	**<0.0001**	0.1300	0.640	0.340	**<0.0001**	0.1700	0.058	0.344	**<0.0001**	0.1600	0.059
		PLWH on ART	0.220	**0.0003**	0.1100		0.180	**0.0040**	0.0880		0.200	**<0.0001**	0.1000	
	**Fucosylated**	PLWoH	-0.230	**0.0004**	-0.0540	0.510	-0.096	0.1270	-0.0220	0.410	-0.250	**<0.0001**	-0.0700	0.130
		PLWH on ART	-0.100	0.0900	-0.0360		0.010	0.8560	0.0070		-0.100	**0.0400**	-0.0300	
	**Agalactosylated**	PLWoH	0.500	**<0.0001**	0.6000	0.670	0.260	**<0.0001**	0.3000	0.750	0.290	**<0.0001**	0.3300	0.160
		PLWH on ART	0.430	**<0.0001**	0.6500		0.290	**<0.0001**	0.3300		0.330	**<0.0001**	0.4300	
	**Terminal galactosylated**	PLWoH	-0.510	**<0.0001**	-0.5900	0.720	-0.220	**<0.0001**	-0.2300	0.590	-0.310	**<0.0001**	-0.3200	0.260
		PLWH on ART	-0.440	**<0.0001**	0.6300		-0.270	**<0.0001**	-0.2700		-0.340	**<0.0001**	-0.4000	
	**Sialylated**	PLWoH	-0.439	**<0.0001**	-0.2800	0.340	-0.300	**<0.0001**	-0.1700	0.960	-0.270	**<0.0001**	-0.1500	0.380
		PLWH on ART	-0.357	**<0.0001**	-0.2400		-0.320	**<0.0001**	-0.1900		-0.312	**<0.0001**	-0.1900	

■ *N*-acetylglucosamine ▲ Fucose ● Mannose ● Galactose ◆ Sialic acid

Two-tailed Spearman's rank correlations.

Supplementary Table 4. Characteristics of participants in the longitudinal analysis presented in Supplementary Figure 4.

			Visit 1		Visit 2		Visit 3		Visit 4		Visit 5		Visit 6		Visit 7		Visit 8	
			PLWoH	PLWH on ART	PLWoH	PLWH on ART	PLWoH	PLWH on ART	PLWoH	PLWH on ART	PLWoH	PLWH on ART	PLWoH	PLWH on ART	PLWoH	PLWH on ART	PLWoH	PLWH on ART
Women	n		23	23	23	23	23	23	23	23	23	23	23	23	18	21	8	0
	Age (years, median IQR)		49 (5.5)	50 (5.5)	50 (6)	51 (5)	51 (6)	53 (5.5)	52 (5.5)	54 (6)	54 (5.5)	56 (5.5)	56 (5.5)	57 (6)	56 (5.8)	58 (5)	59 (6.5)	-
	BMI (median, IQR)		30 (8.2)	30 (8.2)	30 (8.7)	30 (7.6)	28 (9)	30.8 (8.8)	28 (9.6)	30 (11.5)	28 (8.3)	30 (10)	29 (9.2)	29.5 (10.8)	29 (9.5)	30.3 (7.5)	30 (8.8)	-
	Blood CD4 count (cells/mm^3)			774 (236)		812 (406)		797 (310)		821 (394)		837 (402)		750 (433)		745 (285)		-
	Plasma viral load (copies/ml)		-	<50	-	<50	-	<50	-	<50	-	<50	-	<50	-	<50	-	-
	Nadir CD4 count (cells/mm^3)		-	209 (166.5)	-	-	-	-	-	-	-	-	-	-	-	-	-	-
	Race	White, non-Hispanic	4	4	-	-	-	-	-	-	-	-	-	-	-	-	-	-
		Black, Hispanic	17	17	-	-	-	-	-	-	-	-	-	-	-	-	-	-
		Other Hispanic	2	2	-	-	-	-	-	-	-	-	-	-	-	-	-	-
Men	n		24	24	24	24	24	24	24	24	24	24	23	24	6	5	1	0
	Age (years, median IQR)		49 (4)	49 (4)	50 (4)	50 (4)	52 (4)	52 (4)	54 (4)	54 (3)	55 (4)	56 (4)	57 (4)	59 (4)	57 (2)	58 (2)	59 (0)	-
	BMI (median, IQR)		24 (4)	25 (2.8)	25 (3.7)	25 (3.2)	25 (4.3)	24 (4.1)	24 (5.1)	25 (4.2)	24 (3.9)	25 (3.9)	24 (3.7)	25 (4.3)	26 (1.9)	25 (3.6)	26 (0)	-
	Blood CD4 count (cells/mm^3)		898 (368)	683 (402)	907 (301)	711 (357)	958 (352)	720 (217)	946 (234)	728 (222)	890 (177)	713 (263)	954 (184)	749 (391)	812 (290)	680 (448)	970 (0)	-
	Plasma viral load (copies/ml)		-	<50	-	<50	-	<50	-	<50	-	<50	-	<50	-	<50	-	-
	Nadir CD4 count (cells/mm^3)		540 (138)	272 (181)	-	-	-	-	-	-	-	-	-	-	-	-	-	-
	Race	White, non-Hispanic	22	22	-	-	-	-	-	-	-	-	-	-	-	-	-	-
		Black, non-Hispanic	2	2	-	-	-	-	-	-	-	-	-	-	-	-	-	-

Supplementary Table 5. Machine learning models based on IgG glycans and/or inflammatory markers.

Sex		Lasso model					Training model data					Testing model				
		Selected predictor(s)	n	R^2	Adjusted R^2	MSE	n	MSE	SD	Low 95% CI	Upper 95% CI	n	MSE	SD	Low 95% CI	Upper 95% CI
Men	**IgG glycans (model 1)**	A2 + FA2B + FA2BG1 + A2G2S2	253	0.23	0.21	44.19	202 (80%)	44.22	2.22	39.88	48.57	51 (20%)	45.46	9.27	27.30	63.62
	**IgG glycans (model 1)**	A2 + FA2B + FA2BG1 + A2G2S2	100	0.36	0.34	38.68										
	**IgG glycans (model 2)**	A2 + FA2B + FA2BG1	253	0.19	0.19	45.98	202 (80%)	45.63	2.26	41.20	50.05	51 (20%)	48.27	9.09	30.45	66.09
	**IgG glycans (model 2)**	A2 + FA2B + FA2BG1	100	0.26	0.23	45.26										
	**Inflammatory markers***	CXCL9 + Eotaxin	100	0.02	0.003	59.42										
	**IgG glycans (model 1) + Inflammatory markers**	A2 + FA2B + FA2BG1 + A2G2S2 + CXCL9 + Eotaxin	100	0.37	0.33	38.02										
	**IgG glycans (model 2) + Inflammatory markers**	A2 + FA2B + FA2BG1 + CXCL9 + Eotaxin	100	0.29	0.25	43.28										
Women	**IgG glycans**	FA2G2S1 + FA2G2	235	0.31	0.31	36.25	188 (80%)	36.40	1.78	32.91	39.88	47 (20%)	36.25	7.27	22.00	50.49
	**IgG glycans**	A1F + G2F	100	0.25	0.23	32.16										
	**Inflammatory markers***	CXCL9 + Eotaxin	100	0.23	0.22	32.82										
	**IgG glycans + Inflammatory markers**	FA2G2S1 + FA2G2 + CXCL9 + Eotaxin	100	0.41	0.38	25.32										

* Pre-selected

MSE = Mean Squared Error

SD = Standard Deviation

Supplementary Table 6. Correlations between inflammatory markers and chronological age.

Inflammatory marker	WLWoH				WLWH on ART				MLWoH				MLWH on ART			
	n	Spearman's *rho*	*P* value	FDR	n	Spearman's *rho*	*P* value	FDR	n	Spearman's *rho*	*P* value	FDR	n	Spearman's *rho*	*P* value	FDR
**Fractalkine**	100	0.225	**0.024**	0.106	100	0.039	0.698	0.870	100	0.131	0.192	0.534	100	0.263	**0.008**	0.060
**IFN-α2a**	64	0.059	0.643	0.884	50	-0.003	0.982	0.982	97	0.108	0.291	0.640	94	0.129	0.217	0.284
**IL-12p70**	89	0.105	0.325	0.651	87	0.143	0.186	0.371	91	0.026	0.806	0.934	84	0.174	0.113	0.253
**IL-2**	85	-0.006	0.957	0.957	73	0.163	0.168	0.371	89	0.139	0.194	0.534	86	0.287	**0.007**	0.060
**IL-4**	73	0.035	0.769	0.957	59	-0.035	0.792	0.918	88	-0.030	0.782	0.934	85	0.194	0.076	0.212
**IL-5**	92	-0.013	0.899	0.957	72	-0.044	0.712	0.870	92	-0.004	0.967	0.996	91	0.157	0.138	0.253
**IP-10**	100	0.049	0.626	0.884	100	0.187	0.062	0.227	100	0.203	**0.043**	0.315	100	0.240	**0.016**	0.090
**MCP-2**	100	0.177	0.078	0.215	100	0.087	0.390	0.613	100	-0.001	0.996	0.996	100	-0.030	0.766	0.802
**MIP-1α**	100	0.068	0.501	0.884	98	0.019	0.851	0.936	100	-0.061	0.549	0.805	99	0.231	**0.022**	0.095
**SDF-1a**	100	0.200	**0.046**	0.150	100	0.073	0.471	0.691	100	0.063	0.533	0.805	100	0.137	0.173	0.253
**Eotaxin**	100	0.393	**5.26E-05**	**0.001**	100	0.323	**0.001**	**0.012**	100	0.118	0.241	0.589	100	-0.283	**0.004**	0.060
**IFN-β**	36	0.225	0.186	0.455	41	-0.360	**0.021**	**0.115**	38	-0.242	0.143	0.523	48	-0.151	0.307	0.356
**IFN-γ**	93	0.067	0.525	0.884	96	0.098	0.344	0.583	100	0.181	0.071	0.389	100	0.116	0.249	0.304
**IL-10**	92	0.006	0.952	0.957	96	0.157	0.127	0.315	99	0.082	0.420	0.805	98	0.179	0.077	0.212
**IL-1β**	85	-0.008	0.944	0.957	80	0.180	0.111	0.315	91	-0.164	0.119	0.523	94	0.158	0.127	0.253
**IL-21**	22	0.510	**0.015**	0.084	15	0.309	0.263	0.482	65	0.034	0.789	0.934	72	0.165	0.165	0.253
**IL-6**	100	0.198	**0.048**	0.150	100	-0.055	0.586	0.806	100	-0.049	0.628	0.864	99	0.125	0.220	0.284
**Leptin**	100	-0.049	0.627	0.884	98	-0.154	0.129	0.315	100	-0.075	0.460	0.805	100	0.059	0.560	0.616
**CXCL9**	100	0.385	**7.58E-05**	**0.001**	100	0.359	**2.42E-04**	**0.005**	100	0.342	**0.001**	**0.011**	100	0.139	0.168	0.253
**TNF-α**	100	0.285	**0.004**	**0.030**	99	0.207	**0.040**	0.175	100	0.214	**0.033**	0.315	100	0.158	0.115	0.253
**CD14**	100	0.118	0.240	0.529	99	-0.011	0.914	0.957	100	-0.004	0.966	0.996	98	0.023	0.821	0.821
**CD163**	100	0.014	0.892	0.957	99	0.289	**0.004**	0.027	100	0.067	0.506	0.805	98	0.200	**0.049**	0.179

Spearman's rank correlation and FDR values were calculated using the Benjamini-Hochberg procedure to correct for multiple comparisons.

Supplementary Table 7. Baseline characteristics of the subclinical atherosclerosis study participants in studies presented in Figure 5.

		PLWoH controls (n=22)	PLWoH cases (n=22)	*P* value (PLWoH, cases vs controls)	PLWH controls (n=34)	PLWH cases (n=34)	*P* value (PLWH, cases vs controls)
**Age (years; mean, range)**		57.4 (48-71)	57.86 (49-65)	0.654	51.35 (43-63)	51.79 (44-62)	0.653
**BMI (mean, range)**		25.45 (20.1-33.2)	28.66 (21-38.3)	0.066	26.03 (17.7-34)	24.94 (17.4-33.6)	0.302
**CD4 count (cells/mm^3; mean, range)**		846.41 (288-1565)	982.9 (551-1809)	0.99	591.94 (103-1228)	623.87 (205-1255)	0.62
**Nadir CD4 (cells/mm^3; mean, range)**		-	-	-	282.88 (8-733)	227.58 (0-560)	0.19
**Systolic Blood Pressure (mm Hg; mean, range)**		123.76 (103-148)	130.71 (110-151)	0.057	126.42 (108-164)	124.09 (100-148)	0.472
**Diastolic Blood Pressure (mm Hg; mean, range)**		75.89 (62-95)	81.95 (66-108)	**0.041**	79.63 (65-91)	76.45 (61-101)	0.069
**Fasting glucose (mg/dL; mean, range)**		93.45 (68-133)	113 (72-247)	**0.021**	104.6 (78-277)	106.93 (80-252)	0.626
**Total cholesterol (mg/dL; mean, range)**		185.86 (138-259)	202.31 (133-320)	0.265	190.23 (115-294)	186.63 (127-269)	0.749
**High Density Lipoprotein (mg/dL; mean, range)**		55.99 (38.5-90.8)	49.44 (29-73.7)	0.213	49.82 (22.9-101)	45.18 (25-82.6)	0.35
**Triglycerides (mg/dL; mean, range)**		88.77 (23-199)	168.81 (45-344)	**<0.0001**	143.6 (43-425)	141.36 (47-304)	0.958
**Low Density Lipoprotein (mg/dL; mean, range)**		112.18 (56-166)	119.09 (52-210)	0.639	113.87 (22-218)	113.72 (65-202)	0.818
**Framingham coronary heart disease 10 yr risk (%; mean, range)**		7.36 (2-18)	13.45 (3-27)	**0.004**	8.44 (3-47)	9.14 (2-33)	0.27
**Framingham hard coronary heart disease, 10 yr risk (%; mean,**		6.95 (1-12)	13 (3-30)	**0.006**	8 (1-30)	8.11 (1-25)	0.771
**ACC/AHA risk estimate (mean, range)**		0.08 (0.027-0.225)	0.12 (0.027-0.218)	**0.014**	0.069 (0.012-0.291)	0.077 (0.007-0.339)	0.5
**Cumulative years of ART use (mean, range)**		-	-	-	7.91 (0-15.41)	9.3 (0.05-14.25)	0.14
**Viral suppression (%)**	**Supressed**	-	-	-	91.2%	82.4%	0.48
	**Non-supressed**	-	-		8.8%	17.6%	
**Diagnosed with AIDS (%)**	**No AIDS Dx**	-	-	-	85.3%	82.4%	>0.9
	**AIDS Dx**	-	-		14.7%	17.6%	
**Statin use (%)**	**No**	68.2%	45.5%	0.22	61.8%	61.8%	>0.9
	**Yes**	31.8%	54.5%		38.2%	38.2%	
**Aspirin use (%)**	**No**	27.3%	27.3%	>0.9	58.8%	58.8%	>0.9
	**Yes**	72.7%	72.7%		41.2%	41.2%	
**Diabetes status (%)**	**No**	95.5%	77.3%	0.185	79.4%	73.5%	0.76
	**Yes**	4.5%	22.7%		17.6%	20.6%	
	**Insufficient data**	0.0%	0.0%		2.9%	5.9%	
**Cancer diagnosis (%)**	**No Cancer Dx**	95.5%	81.8%	0.34	82.4%	82.4%	>0.9
	**Cancer Dx**	4.5%	18.2%		17.6%	17.6%	
**Smoking Status At Visit (%)**	**Never smoked**	36.4%	22.7%	-	50.0%	17.6%	-
	**Former smoker**	59.1%	59.1%		32.4%	52.9%	
	**Current smoker**	4.5%	18.2%		17.6%	23.5%	
	**Unknown**	0.0%	0.0%		0.0%	5.9%	
**Race (%)**	**White, non-Hispanic**	81.8%	81.8%	-	61.8%	61.8%	-
	**White, Hispanic**	4.5%	4.5%		2.9%	2.9%	
	**Black, non-Hispanic**	13.6%	13.6%		20.6%	20.6%	
	**Black, Hispanic**	0.0%	0.0%		8.8%	0.0%	
	**Other**	0.0%	0.0%		2.9%	0.0%	
	**Other Hispanic**	0.0%	0.0%		2.9%	14.7%	

Two-tailed Mann-Whitney U tests were used for continuous variables, and Fisher's exact tests were used for categorical values.

Supplementary Table 8. Plasma markers of inflammation in of the subclinical atherosclerosis study participants in studies presented in Figure 5.

	PLWoH controls (n=22)	PLWoH cases (n=22)	PLWH controls (n=34)	PLWH cases (n=34)	ANOVA/ KW Test
	Mean (SD) pg/ml	Mean (SD) pg/ml	Mean (SD) pg/ml	Mean (SD) pg/ml	*P-* value
**sCD163**	548 (188.9)	600.3 (213.7)	683.3 (391.7)	883.9 (630.3)	**0.020**
**sCD14**	1633137 (367471)	1580028 (348586)	1900147 (549256)	2011467 (470251)	**0.001**
**Fractalkine**	5929.8 (1559.5)	5374.9 (1025.3)	6811.5 (2388.3)	6273.8 (1758.3)	**0.034**
**IL10**	0.25 (0.24)	0.53 (0.89)	0.33 (0.35)	0.43 (0.57)	0.447
**IL1β**	0.81 (2)	0.29 (0.53)	0.2 (0.15)	0.24 (0.39)	0.996
**IL6**	0.76 (0.33)	1.1 (0.95)	1.27 (1.941)	1.59 (2.67)	0.435
**MCP2**	21.04 (7.38)	22.86 (5.96)	28.09 (13.27)	30.43 (21.94)	**0.007**
**MIP1α**	18 (10.31)	17.89 (6.343)	18.37 (10.38)	46.08 (133.72)	0.908
**SDF1α**	734.5 (415.4)	846.1 (545.8)	761.5 (443.1)	839.1 (254.2)	0.216
**TNFα**	1.7 (1.6)	1.53 (0.79)	1.73 (1.3)	2.68 (3.63)	0.632
**TGFβ1**	7397.7 (6398.9)	6691.3 (6832.1)	7299.5 (8438.1)	9173.2 (10822.5)	0.490
**TGFβ2**	36.84 (25.2)	37.51 (29.7)	45.27 (54.9)	66.64 (82.3)	0.124
**TGFβ3**	2.73 (1.47)	2.86 (1.55)	3.06 (2.56)	3.092 (2.35)	0.810

ANOVA/ Kruskal–Wallis test Tests

Supplementary Table 9. Characteristics of participants in studies presented in Figure 6.

		Cases		Control		*P* value
		n (%)	Median, IQR	n (%)	Median, IQR	
**n**		10		13		
**Race**	Black/African-American	5 (50)	-	6 (46.2)	-	
	Not Hispanic or Latino	1 (10)	-	1 (7.7)	-	
	White/Caucasian	4 (40)	-	6 (46.2)	-	
**Blood CD4 count (cells/mm^3)**		-	497 (334)	-	873 (328)	**0.0025**
**Plasma viral load (HIV copies/ml)**		-	< 50	-	< 50	
**Nadir CD4 count (cells/mm^3)**		-	153 (158)	-	531 (290)	**0.0002**

Two-tailed Mann-Whitney U tests

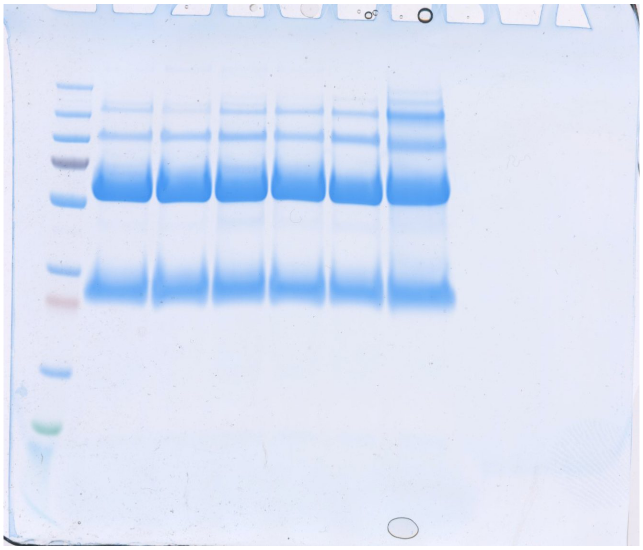

Uncropped image of the gel in Supplementary Figure 1c.
