# Supplementary material for: Immunoglobulin G N-glycan markers of accelerated biological aging during chronic HIV infection
Source: Nat Commun. 2024 Apr 10;15:3035. doi: 10.1038/s41467-024-47279-4 (PMC11006954; doi:10.1038/s41467-024-47279-4)
Supplement: Supplementary file 3 — Reporting Summary [file 41467_2024_47279_MOESM3_ESM.pdf]

nature portfolio

Corresponding author(s): Mohamed Abdel-Mohsen

Last updated by author(s): 11/26/2023

Reporting Summary

Nature Portfolio wishes to improve the reproducibility of the work that we publish. This form provides structure for consistency and transparency in reporting. For further information on Nature Portfolio policies, see our Editorial Policies and the Editorial Policy Checklist.

Statistics

For all statistical analyses, confirm that the following items are present in the figure legend, table legend, main text, or Methods section.

n/a	Confirmed	
☐	☒	The exact sample size (*n*) for each experimental group/condition, given as a discrete number and unit of measurement
☐	☒	A statement on whether measurements were taken from distinct samples or whether the same sample was measured repeatedly
☐	☒	The statistical test(s) used AND whether they are one- or two-sided<br>*Only common tests should be described solely by name; describe more complex techniques in the Methods section.*
☐	☒	A description of all covariates tested
☐	☒	A description of any assumptions or corrections, such as tests of normality and adjustment for multiple comparisons
☐	☒	A full description of the statistical parameters including central tendency (e.g. means) or other basic estimates (e.g. regression coefficient) AND variation (e.g. standard deviation) or associated estimates of uncertainty (e.g. confidence intervals)
☐	☒	For null hypothesis testing, the test statistic (e.g. *F*, *t*, *r*) with confidence intervals, effect sizes, degrees of freedom and *P* value noted<br>*Give P values as exact values whenever suitable.*
☒	☐	For Bayesian analysis, information on the choice of priors and Markov chain Monte Carlo settings
☒	☐	For hierarchical and complex designs, identification of the appropriate level for tests and full reporting of outcomes
☐	☒	Estimates of effect sizes (e.g. Cohen's *d*, Pearson's *r*), indicating how they were calculated

Our web collection on statistics for biologists contains articles on many of the points above.

Software and code

Policy information about availability of computer code

Data collection	N-glycans data were collected using the GlycanAssure Data Analysis Software (version 2.0). Flow Cytometry data were analyzed using FACSDiva (version 8.0.1)
Data analysis	GraphPad Prism (version 9), R (version 4.0.3), FlowJo (version 10.7.01), and GlycanAssure Data Analysis Software (version 2.0).

For manuscripts utilizing custom algorithms or software that are central to the research but not yet described in published literature, software must be made available to editors and reviewers. We strongly encourage code deposition in a community repository (e.g. GitHub). See the Nature Portfolio guidelines for submitting code & software for further information.

Data

Policy information about availability of data

All manuscripts must include a data availability statement. This statement should provide the following information, where applicable:

- Accession codes, unique identifiers, or web links for publicly available datasets
- A description of any restrictions on data availability
- For clinical datasets or third party data, please ensure that the statement adheres to our policy

The authors declare that data supporting the findings of this study are available within the paper and its supplementary information files. In addition, Mass spectrometry proteomic data were deposited to ProteomeXchange via the PRIDE database (project accession: PXD046510; DOI: 10.6019/PXD046510). Capillary electrophoresis data were uploaded to Zenodo (DOI: 10.5281/zenodo.10553464). Single cell RNAseq data were deposited to Gene Expression Omnibus (GEO) with accession # GSE254483. Access to individual-level clinical data from the MACS/WIHS Combined Cohort Study Data (MWCCS) may be obtained upon review and

approval of a MWCCS concept sheet. Links and instructions for online concept sheet submission are on the study website.

Research involving human participants, their data, or biological material

Policy information about studies with human participants or human data. See also policy information about sex, gender (identity/presentation), and sexual orientation and race, ethnicity and racism.

Reporting on sex and gender	We used samples from both men and women, as assigned at birth in this study. Statistical analysis was conducted separately by sex.
Reporting on race, ethnicity, or other socially relevant groupings	We used samples from multiple races and considered it in the statistical analysis.
Population characteristics	We analyzed the IgG glycomes of 254 women with HIV (WWH) on suppressive ART for at least five years with undetectable HIV viral load, weight less than 300 pounds, and with a median CD4 T cell count of 726 cells/mm3. We matched these samples with samples from 235 HIV-negative women matched to the WWH in terms of age, race, and body mass index (BMI) (Table 1). To ensure a wide age range for analysis, we aimed to have a similar number of individuals in each of the following age categories: ≤45, 46-50, 51-55, 56-60, 60-65, and >65 years (Table 1). Using the same criteria, we also analyzed samples from 243 men with HIV (MWH) who were suppressed on ART with a median CD4 T cell count of 698 cells/mm3 and 253 HIV-negative matched controls (Table 1).
Recruitment	Participants were recruited at the MWCCS cohort. The MWCCS is enrolling men and women who are living with HIV, as well as men and women who are at risk for HIV. The MWCCS recruit participants from several study sites, including: Alabama, California, Florida, Georgia, Illinois, Maryland, Mississippi, New York, Ohio, Pennsylvania, and Washington, DC. Study participants recruitment and/or sampling was not part of this study.
Ethics oversight	The study protocols were approved by the Institutional Review Board of The Wistar Institute (IRB protocol 21808309) as well as the Institutional Review Boards of Johns Hopkins University, University of Pennsylvania, University of Georgia, Albert Einstein College of Medicine, Emory University, SUNY Downstate Health Sciences University, University of Miami, University of Pittsburgh, University of North Carolina at Chapel Hill, University of California Los Angeles, Georgetown University Medical Center, University of California San Francisco, University of Alabama at Birmingham, Northwestern University, and Lundquist Institute of Biomedical Research at Harbor-UCLA Medical Center. Written informed consents were obtained from all participants. All human experimentation was conducted per the guidelines set forth by the US Department of Health and Human Services and the authors' respective institutions.

Note that full information on the approval of the study protocol must also be provided in the manuscript.

Field-specific reporting

Please select the one below that is the best fit for your research. If you are not sure, read the appropriate sections before making your selection.

☒ Life sciences ☐ Behavioural & social sciences ☐ Ecological, evolutionary & environmental sciences

For a reference copy of the document with all sections, see nature.com/documents/nr-reporting-summary-flat.pdf

Life sciences study design

All studies must disclose on these points even when the disclosure is negative.

Sample size	For Figures 1, 2, 3, 4, and 8: 254 women and 243 men with HIV on suppressive ART for at least five years. 235 HIV-negative women and 253 HIV-negative men matched controls. For supplementary Figure 4: 622 samples from 94 people living with and without HIV. For Figure 5: 112 men living or not with HIV. For Figure 6: 89 samples from 23 people living with HIV. For Figure 9, 3-4 biological samples.
Data exclusions	No data was excluded from analysis
Replication	all in vitro experiments were done in 3-4 biological replicates and data were consistent between replicates.
Randomization	all analyses were performed randomly to avoid batch to batch effects
Blinding	Investigator were blinded on samples clinical data while running the experiments and Statistical analyses were done separately by the statistical team at The Wistar Institute.

Reporting for specific materials, systems and methods

We require information from authors about some types of materials, experimental systems and methods used in many studies. Here, indicate whether each material, system or method listed is relevant to your study. If you are not sure if a list item applies to your research, read the appropriate section before selecting a response.

Materials & experimental systems

n/a	Involved in the study	
☐	☒	Antibodies
☐	☒	Eukaryotic cell lines
☒	☐	Palaeontology and archaeology
☒	☐	Animals and other organisms
☒	☐	Clinical data
☒	☐	Dual use research of concern
☒	☐	Plants

Methods

n/a	Involved in the study	
☒	☐	ChIP-seq
☐	☒	Flow cytometry
☒	☐	MRI-based neuroimaging

Antibodies

Antibodies used

the HIV antibody 10-1074 for the glycoengineering experiments (received from Rockefeller University). The anti-HIV-1 core antigen (p24)-RD1 (Beckman Coulter catalog # 6604667, clone KC57) for the ADCC experiments. The following antibodies from BioLegend were used for the single-cell CITE-Seq experiments: anti-human CD14 (catalog# 367145, clone 63D3), anti-human CD16 (catalog# 302063, clone 3G8), anti-human CD19 (catalog# 302263, clone HIB19), anti-human CD20 (catalog# 302361, clone 2H7), anti-human CD27 (catalog# 302851, clone O323), anti-human CD3 (catalog# 300477, clone UCHT1), anti-human CD38 (catalog# 356639, clone HB-7), anti-human CD4 (catalog# 300565, clone RPAT-4), anti-human CD56 (catalog# 362561, clone 5.1H11), anti-human HLA-DR (catalog# 307661, clone L243), anti-human CD45RA (catalog# 304161, clone HI100), and anti-human CD8 (catalog# 344757, clone SK1).

Validation

The 10-1074 antibody is being used for clinical trials and undergoes extensive validation. Antibodies from BioLegend andBeckman Coulter are validated by the manufactures.

Eukaryotic cell lines

Policy information about cell lines and Sex and Gender in Research

Cell line source(s)

CEM.NKR CCR5+ Luc+ cells: Human T Lymphoblastic Leukemia (NIH AIDS reagent catalog #ARP-5198)
THP-1: human monocyte isolated from peripheral blood from an acute monocytic leukemia patient.
HUT78: Human T-Cell Lymphoma (NIH AIDS reagent catalog #ARP-89
TZM-bl cells: indicator cell line that is highly sensitive to infection with diverse isolates of HIV-1 and enables quantitative analysis of HIV infection using either β-galactosidase or luciferase as a reporter. (NIH AIDS reagent catalog # ARP-8129

Authentication

None of these cells lines were authenticated

Mycoplasma contamination

Cells were not tested for mycoplasma

Commonly misidentified lines
(See ICLAC register)

No misidentified lines

Plants

Seed stocks

Report on the source of all seed stocks or other plant material used. If applicable, state the seed stock centre and catalogue number. If plant specimens were collected from the field, describe the collection location, date and sampling procedures.

Novel plant genotypes

Describe the methods by which all novel plant genotypes were produced. This includes those generated by transgenic approaches, gene editing, chemical/radiation-based mutagenesis and hybridization. For transgenic lines, describe the transformation method, the number of independent lines analyzed and the generation upon which experiments were performed. For gene-edited lines, describe the editor used, the endogenous sequence targeted for editing, the targeting guide RNA sequence (if applicable) and how the editor was applied.

Authentication

Describe any authentication procedures for each seed stock used or novel genotype generated. Describe any experiments used to assess the effect of a mutation and, where applicable, how potential secondary effects (e.g. second site T-DNA insertions, mosiacism, off-target gene editing) were examined.

Flow Cytometry

Plots

Confirm that:

- [x] The axis labels state the marker and fluorochrome used (e.g. CD4-FITC).
- [x] The axis scales are clearly visible. Include numbers along axes only for bottom left plot of group (a 'group' is an analysis of identical markers).
- [x] All plots are contour plots with outliers or pseudocolor plots.
- [x] A numerical value for number of cells or percentage (with statistics) is provided.

Methodology

Sample preparation	Cell line were collected after assay and washed with PBS. Antibodies for surface staining were added and incubated for 30min protected from light. After surface staining, when intracellular staining is needed, cells were fixed with 100 µL of Cytofix/Cytoperm for 15 minutes at room temperature and permeabilized with 1X Perm/wash buffer. The cells were then incubated for 30 minutes with intracellular staining. After this incubation, the cells were washed, resuspended, and at least 100,000 events were acquired. If there is no intracellular staining, cells were just fixed as described and resuspended to flow collection.
Instrument	BD Biosciences FACS Symphony
Software	Data was collect on BD Diva and analyzed using FlowJo
Cell population abundance	No post-sorting fractions.
Gating strategy	For ADCP: FSC and SSC gating to remove debris and other events of non-interest, followed by FSC-H and FSC-A to keep single cells only. From single cell gate, using SSC and FITC to gate FITC positives cells (cells containing the beads).<br>For ADCD: FSC and SSC gating to remove debris and other events of non-interest, followed by FSC-H and FSC-A to keep single cells only. From single cell gate, examine calcualte MFI of C3a.<br>For the p24 intracellular staining: FSC and SSC gating to select CEM cells, followed by FSC-H and FSC-A to keep single cells only. From single cell gate, using SSC and PE to select HIV p24+ cells

- [x] Tick this box to confirm that a figure exemplifying the gating strategy is provided in the Supplementary Information.
